# Morphologic and phenotypic characteristics of myocarditis in two pigs infected by foot-and mouth disease virus strains of serotypes O or A

**DOI:** 10.1186/s13028-014-0042-6

**Published:** 2014-07-12

**Authors:** Carolina Stenfeldt, Juan M Pacheco, Manuel V Borca, Luis L Rodriguez, Jonathan Arzt

**Affiliations:** 1 Plum Island Animal Disease Center, Foreign Animal Disease Research Unit, Agricultural Research Service, United States Department of Agriculture, Greenport 11944, NY, USA; 2 Oak Ridge Institute for Science and Education, PIADC Research Participation Program, Oak Ridge 37831, TN, USA

**Keywords:** Foot-and-mouth disease, Myocarditis, Virus, Pig, Pathology

## Abstract

Myocarditis is often cited as the cause of fatalities associated with foot-and-mouth disease virus (FMDV) infection. However, the pathogenesis of FMDV-associated myocarditis has not been described in detail. The current report describes substantial quantities of FMDV in association with a marked mononuclear inflammatory reaction, interstitial edema and cardiomyocyte degeneration in the myocardium of two pigs that died during acute infection with either of two different strains of FMDV. Despite similar clinical progression, there was a marked variation in morphological characteristics of myocarditis with a significant difference in intensity of myocardial inflammation between the two cases. Phenotypic characterization of leukocyte populations revealed that in both cases, the inflammatory infiltrate consisted mainly of combinations of CD172a+, CD163+ and CD44+ cells, with a distinct subset of CD8+ cells, but with consistent lack of detection of CD3+ and CD21+ cells. This suggests that the FMDV-associated acute myocardial inflammation in the two observed cases consisted mainly of leukocytes of monocyte lineage, with a distinct population of CD8+ cells which, based on lack of CD3 detection in serial sections, are likely to represent NK cells.

## Background

Foot-and-mouth disease (FMD) is a highly contagious vesicular infection of cloven-hoofed animals caused by FMD virus (FMDV), a single stranded positive sense RNA virus of the Picornavirus family. FMD is generally a disease that causes high morbidity and low mortality. However, there are sporadic reports of FMD outbreaks with unusually high mortality rates [[1],[2]], with fatalities often being attributed FMDV-associated myocarditis (as reviewed by [[3]]). Investigations based on specimens obtained from field outbreaks [[2],[4]], as well as experimental studies [[5],[6]], have confirmed a relationship between acute FMDV infection and fatal myocarditis in young ruminants and pigs.

Although commonly reported as the cause of fatalities associated with this disease, detailed investigations into the pathogenesis of FMDV myocarditis are lacking. The current report presents virological and histopathological findings in the myocardium of two pigs that died during acute infection with FMDV strains A24 Cruzeiro (case 1) or O1 Campos (case 2). The association between the pigs’ deaths and the presence of FMDV in myocardial tissue samples was confirmed through virus isolation, quantitative reverse transcription polymerase chain reaction (qRT-PCR) and immunomicroscopy. Myocarditic properties of the two FMDV strains were investigated through routine histopathological evaluation in combination with phenotypic characterization of inflammatory infiltrates.

### Case presentation

This report is based on data obtained from two pigs (castrated male Yorkshire pigs of approximately 20 kg) which were associated with separate experimental studies that have been described in detail elsewhere [[7],[8]]. The animals were infected through either direct intra-oropharyngeal (IOP) inoculation (FMDV A24 Cruzeiro, Case 1[[8]]), or through direct contact exposure to previously infected pigs (FMDV O1 Campos, Case 2 [[7]]).

The two pigs developed severe clinical FMD at four (case 1) and two (case 2) days after virus exposure (dpe), with fever (>40°C), lameness and vesiculation at lesion predilection sites (including snout, oral cavity, heel bulbs and coronary bands). Both pigs died with clinical signs of congestive heart failure (respiratory distress and tachycardia) on the morning of the second day of clinical FMD infection (5 dpe for case 1; 3 dpe for case 2). Postmortem examinations of both pigs revealed multifocal regions of pallor on the epicardial surfaces, which extended into the myocardium when sectioned. No other abnormalities were observed.

### Sample collection and analysis

Cardiac tissue samples were harvested during necropsy examinations, and were split into aliquots that were either frozen on liquid nitrogen for virus isolation and qRT-PCR [[9]], embedded in optimal cutting medium (Sakura Finetek, CA) and frozen above a bath of liquid nitrogen for immunomicroscopy [[10]], or fixed in 10% neutral buffered formalin for routine histology. Detection of FMDV antigen and phenotypic characterization of inflammatory cell populations in cryosections was performed by multichannel immunofluorescence (MIF) as previously described [[9],[10]]. Slides were examined with a wide-field, epifluorescence microscope, and images were captured with a cooled, monochromatic digital camera. Images of individual detection channels were adjusted for contrast and brightness and merged in commercially available software (Adobe Photoshop CS6). Monoclonal antibodies used for detection of FMDV antigen and phenotypic characterization of cells are listed in Table 1. Negative control samples were obtained from the myocardium of an uninfected pig. Morphometric leukocyte counts for three pre-defined categories of cells (small mononuclear cells, large mononuclear cells and spindle-shaped cells) were generated by two microscopists that individually analyzed four 40x magnification images taken from affected regions of myocardial sections from both cases. Mean counts for each cell type were compared by students t-test (Graph Pad Quick Calcs; Table 2).

**Table 1:** Antibodies used for multichannel immunofluorescence microscopy

**Antigen**	**Clone**	**Supplier/Product ID**	**Isotype**	**Reactivity**
**FMDV VP1**	6HC4	*in-house*	IgG2b	FMDV serotype A capsid [[16]]
**FMDV VP1**	10GA4	*in-house*	IgG3	FMDV serotype O capsid [[17]]
**Actin**	Acta-1	Lifespan Biosciences/	Rabbit	Actin
**CD163**	2A10-11	Serotec/MCA2311GA	IgG1	Monocytes, macrophages
**CD172a**	74-22-15a	Wasington State University/PG2031	IgG2b	Monocytes, macrophages, granulocytes
**CD44**	P-BAG40A	Wasington State University/PBOV2037	IgG3	Leukocytes
**CD8α**	MIL-12	Serotec/MCA1223	IgG2a	CTLs and NK-cells
**CD3**	SP7	Abcam/ab16669	Rabbit	T-cells
**CD21**	LT21	Abcam/ab1090	IgG1	B-cells

**Table 2:** Histomorphometric analysis

	**Large Mononuclear cells**	**Total Inflammatory cells**
**Case 1**		
Microscopist 1	71.00	88.25
Microscopist 2	83.25	105.00
***Mean/SEM***	***77.13***^**#**^ ***/18.74***	***96.63***^***¥***^ ***/19.16***
**Case 2**		
Microscopist 1	7.50	30.25
Microscopist 2	9.25	27.50
***Mean/SEM***	***8.38***^***#***^ ***/1.61***	***28.88***^***¥***^ ***/2.39***
**Two tailed** ***P*-value**	**0.0026**^**#**^	**0.0035**^**¥**^

Values are means based on cell counts performed in four (pre-determined) image fields for each of the two cases. *P*-values correspond to separate comparisons of the numbers of large mononuclear cells^#^ and the total numbers of inflammatory cells^¥^ across the two cases.

### Case 1

**Figure 1:**
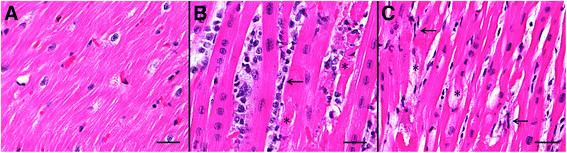
**Histopathological evaluation of myocardium from A) uninfected control pig, B) Case 1, pig that died at 5dpe, FMDV A24 Cruzeiro.** Multifocal myocardial necrosis and fragmentation (*) with interstitial edema and mixed mononuclear infiltrates consisting of small mononuclear cells with sparse to modest cytoplasm, large macrophage-like cells, few spindle cells (←) and scarce neutrophils. **C)** Case 2, Myocardium from pig that died at 3 dpe, FMDV O1 Campos. Multifocal myocardial degeneration and rare necrosis (*) with interstitial edema and cardiomyocyte atrophy and cytoplasmic vacuolization. Mild mixed mononuclear infiltrates consisting of mainly spindle shaped cells with elongated nuclei (←) and small mononuclear cells. Hematoxylin & eosin stain, scale bars = 25 μm.

Serum samples collected one day prior to death (4 dpe) from case 1 (infected with FMDV A24 Cruzeiro) contained 7.62 log_10_ FMDV RNA copies/μl. The highest quantity of viral RNA detected in myocardial tissue samples from this pig was 7.73 log_10_ viral RNA copies/ mg. Virus isolation on LFBK αvβ6 cells [[11]] was positive for both serum and myocardial tissue samples. Myocardium of this pig had multifocal, mixed mononuclear infiltrates that dissected and replaced cardiomyocytes (Figure 1B). Infiltrates consisted of small mononuclear cells with heterochromatic nuclei and modest eosinophilic cytoplasm (presumptive lymphocytes or NKs), large mononuclear cells with euchromatic nuclei (macrophages), and elongated spindle-shaped cells (suspect dendritic cells (DCs)). There were multifocal regions of marked myocardial degeneration and necrosis characterized by cardiomyocyte atrophy, cytoplasmic vacuolation, myofibrillar disruption and mineralization (Figure 1B).

**Figure 2:**
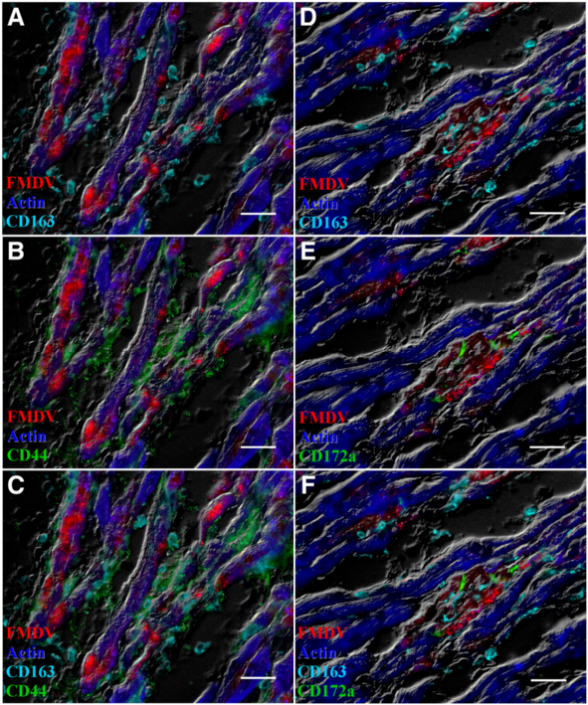
**Myocarditis in two pigs infected with FMDV A24 Cruzeiro (case 1; A-C) or FMDV O1 Campos (case 2; D-F).** In both cases, FMDV VP1 capsid protein co-localizes with actin in cardiomyocytes. Myocardium from case 1 contains marked infiltrates of overlapping populations of CD44+ and CD163+ cells (B-C). Myocardium from case 2 has low numbers of CD172a + and CD163+ cells (E-F). A-C: FMDV A capsid (red), actin (blue), CD163 (turquoise), CD44 (green). D-F: FMDV O capsid (red), actin (blue), CD163 (turquoise), CD 172a (green). Multichannel immunofluorescence. Scale bars = 25 μm.

**Figure 3:**
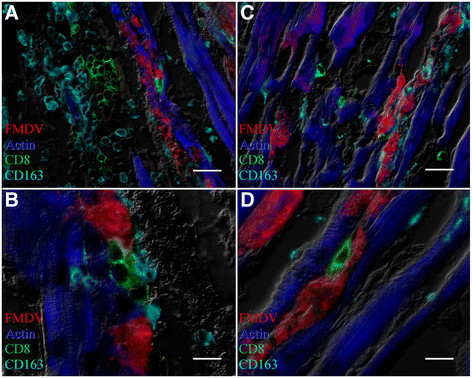
**Myocarditis in two pigs infected with FMDV A24 Cruzeiro (case 1; A-B) or FMDV O1 Campos (case 2; C-D).** Both cases have infiltrates of distinct populations of CD8+ and CD163+ cells which are in close proximity to FMDV infected cardiomyocytes but which do not co-localize with FMDV capsid (red) or actin (blue). On serial sections, there was no detection of CD3 and CD21, indicating a CD8+/CD3-/CD21 –phenotype, consistent with NK-cells. FMDV VP1 capsid protein (red), actin (blue), CD163 (turquoise), CD8 (green). Multichannel immunofluorescence, A&C: scale bars =25 μm. B&D: scale bars = 10 μm.

**Figure 4:**
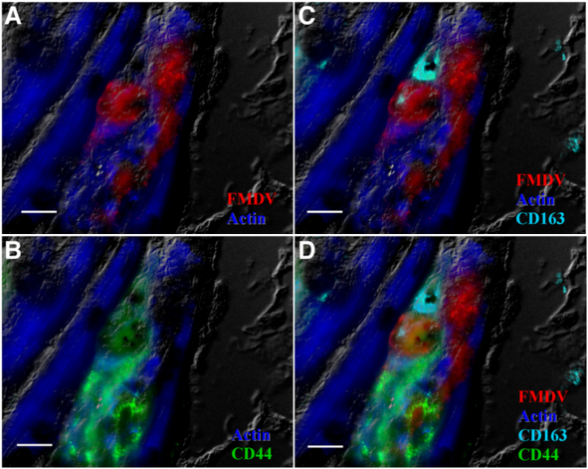
**Myophagocytosis in pig infected with FMDV A24 Cruzeiro (case 1).** A large, mononuclear, CD44+ cell **(B&D)** surrounds an FMDV infected cardiomyocyte. Fragments of FMDV VP1 capsid protein and cardiomyocyte actin are within subcellular structures of the CD44+ cell (presumptive macrophage-lineage; **B&D**). FMDV A capsid (red; **A-D**), actin (blue; **A-D**), CD163 (turquoise; **C&D**), CD44 (green; **B&D**). Multichannel immunofluorescence. Scale bars = 10 μm.

High quantities of FMDV capsid protein VP1 were detected within cardiomyocytes by MIF analysis of cryosections (Figures 2A-C, 3A-B, 4A-D). Phenotypic characterization of inflammatory infiltrate indicated abundant and variably double-positive populations of CD44+ and CD163+ cells within the myocardium (Figure 2A-C). CD44+ cells were observed in close association with infected myocytes and FMDV capsid protein was detected in association with myocardial actin within distinct compartments of some of these cells (Figure 4A-D). There was no detection of CD3+ or CD21+ cells, despite repeated attempts (not shown). However, within areas of inflammatory infiltrates there was a distinct population of CD8+ cells, which did not overlap with expression of CD163 or CD44 (Figure 3A-B). These cells were found in close proximity to infected cardiomyocytes, but with no direct co-localization with FMDV protein (Figure 3A-B).

### Case 2

Serum samples obtained prior to death (3 dpe) from case 2 (infected with FMDV O1 Campos) contained 5.50 log_10_ viral RNA copies/μl, and the highest viral load found in myocardial tissue samples was 6.46 log_10_ RNA copies/mg. Virus isolation on LFBK αvβ6 cells was positive for both serum and tissue samples. Myocardium had regions of myocyte degeneration and interstitial edema, comparable to that observed in case 1 (Figure 1C). Also similar to case 1, there was a mononuclear inflammatory reaction associated with regions of tissue damage, however, there were significantly fewer large mononuclear leukocytes (*P* = 0.0026) and total numbers of inflammatory cells (*P* = 0.0035) compared to case 1 (Table 2; Figure 1B-C). Inflammatory cells consisted mostly of spindle-shaped cells with elongated nuclei, in combination with small mononuclear cells (Figure 1C). The phenotypes of inflammatory cells were similar to those defined for case 1, despite a reduced abundance of cellular infiltrate (Figure 2D-F). For this pig, CD172a was utilized as a 2^nd^ monocyte-lineage marker in combination with CD163. These cells were found in close proximity to infected cardiomyocytes, although, in contrast to observations in tissue sections from case 1, myophagocytic activity could not be clearly demonstrated. Similar to case 1, there was a distinct population of CD8+ cells (Figure 3C-D) without concurrent detection of CD3 or CD21 in serial sections.

## Discussion

The current report presents a descriptive investigation of the myocarditic properties of FMDV, based on analysis of myocardial tissue samples from two pigs that died during acute infection with FMDV A24 Cruzeiro or FMDV O1 Campos. There was a difference in the time elapsed from inoculation to death between the two cases (3 and 5 days respectively). However, both pigs died during the second day of clinical FMD. There are several potential contributors to the differences between the cases including variability of the viral strains used, host immune responses, and manner of exposure to FMDV (IOP inoculation vs. contact exposure). High levels of FMDV were detected by qRT-PCR and virus isolation in the myocardium of both pigs. For both pigs, myocardial levels of FMDV RNA exceeded those measured in serum although there was no serum available for analysis from the day of death for case 1. Histopathological evaluation of tissue sections from both cases indicated comparable extents of myocardial tissue damage, although with a substantially greater quantity of inflammatory cells in samples from the pig infected by FMDV A24 Cruzeiro. Since the cardiomyocyte degeneration is present with and without inflammation, this finding suggests that a substantial portion of the tissue damage may be a direct consequence of virus replication rather than a result of the host-response to infection. There was also a distinction in the morphology of inflammatory cells, with a greater abundance of large macrophage-like cells in case 1, in comparison to a relatively larger proportion of elongated cells (suspect dendritic cells) in case 2 (Figure 1B-C ). This indicates a continuum of potential cellular responses in FMDV-induced myocarditis; however, the causality of the variation in these cases was not determined in the current study.

The phenoptypic composition of the inflammatory infiltrates, as determined by the detection of specific leukocyte cell surface markers, was comparable across both cases. A large proportion of inflammatory cells present in myocardial sections from both cases were CD163+, which is consistent with both macrophages and tissue DCs [[12]]. However, the morphology of these presumptive monocyte-lineage cells varied across the two cases, with predominantly large mononuclear cells in case 1 and small elongated cells in case 2. The combinations of cell surface markers used for characterization of inflammatory cells do not enable clear distinction between DCs and cells of macrophage lineage. The overall findings suggest that the leukocyte populations present in both cases, were of similar histogenesis, but of different stages of maturation and/or activation. It was not possible to use the exact same combinations of cell markers for detection of cells of monocyte lineage across the two FMDV serotypes because of isotype differences in antibodies utilized for FMDV detection, (Table 1). Preliminary studies indicated that a large proportion of inflammatory cells in the myocardium of case 1 were also CD172a+, and that the distribution of these cells was similar to that of CD44+ (not shown). Phenotypic characterization of the inflammatory infiltrates in these two cases was comparable to reports of FMDV associated myocarditis in lambs [[2]], although, in contrast to findings in this current study, the previous publication also reported detection of lymphocytes and plasma cells in myocardial sections.

There was a consistent lack of detection of CD3 and CD21 expression in inflammatory infiltrates, suggesting an absence of B- and T- lymphocytes, which may be related to the very early stages of infection at which these pigs died (3–5 days after infection). The CD8+/CD3-/CD21- phenotype, small mononuclear cells detected in myocardial infiltrates of both cases described herein is consistent with NK-cells [[13]]. The large quantity of these cells, particularly in case 1 suggests that NK-cells are involved in the pathogenesis of acute FMDV myocarditis in pigs. CD8+ cells were found in close proximity to FMDV-infected myocytes, but without intracellular localization of FMDV protein. This contrasts the colocalization of FMDV and actin within intracellular compartments of CD44+ cells (myophagocytosis, Figure 4). These observations are consistent with the cytotoxic functions of NK-cells versus the phagocytic role of macrophages.

Variation in the intensity of myocardial inflammation between different virus strains has previously been reported for Cocksackie B virus, a related virus within *Picornaviridae* [[14]]. Previous descriptions of the characteristics of myocardial injury in pigs caused by encephalomyocarditis virus (EMCV), another Picornavirus, are strikingly similar to the current findings [[15]], suggesting mechanistic similarities between the two viruses and/or the associated host responses. A previous study investigating the pathogenesis of EMCV in pigs described a lympho-histocytic inflammatory response in infected myocardium, which increased progressively from 6 to 30 hours post infection [[15]]. This suggests that the difference in inflammatory intensity of the two pigs of the current study, may indicate that these two cases represents different phases of infection. However, the differences observed between the two cases may be a result of different characteristics of the two FMDV strains with which the pigs were infected.

## Conclusions

Overall, the two cases of FMDV-associated myocarditis described herein had exceedingly similar clinical and pathological characteristics. There was substantial histomorphologic variation between the cases, but the mechanistic differences underlying this disparity could not be determined in this purely descriptive analysis. There is a need for more comprehensive studies to further investigate the pathogenesis of FMDV-associated myocarditis. Analysis of specific host-virus interactions involved in the onset and progression of myocardial injury, including comparative studies of related picornaviruses with more consistent cardiotropism, could help to further elucidate the mechanisms of this atypical, yet important variation of FMDV pathogenesis.

## Competing interests

The authors declare that they have no competing interests.

## Authors’ contributions

CS planned and performed immunofluorescence assays and imaging, executed the animal experiment with FMDV A and drafted the manuscript. JP executed the animal experiment with FMDV O, oversaw laboratory analyses, and edited the manuscript. MB & LR contributed to study design and provided critical revision of the manuscript. JA designed and coordinated the studies, revised manuscript. All authors read and approved the manuscript.
